# A novel visual ranking system based on arterial spin labeling perfusion imaging for evaluating perfusion disturbance in patients with ischemic stroke

**DOI:** 10.1371/journal.pone.0227747

**Published:** 2020-01-24

**Authors:** Sangjoon Lee, Dong Woo Park, Tae Yoon Kim, Dong Sun Kim, Ji Young Lee, Young-Jun Lee, Chun Ki Kim

**Affiliations:** 1 Department of Radiology, Hanyang University College of Medicine, Hanyang University Guri Hospital, Guri, Republic of Korea; 2 Department of Radiology, Hanyang University College of Medicine, Hanyang University Hospital, Seoul, Republic of Korea; 3 Department of Nuclear Medicine, Hanyang University College of Medicine, Hanyang University Hospital, Seoul, Republic of Korea; Universitatsklinikum Freiburg, GERMANY

## Abstract

We developed a visual ranking system by combining the parenchymal perfusion deficits (PPD) and hyperintense vessel signals (HVS) on arterial spin labeling (ASL) imaging. This study aimed to assess the performance of this ranking system by correlating with subtypes classified based on dynamic susceptibility contrast (DSC) imaging for evaluating the perfusion disturbance observed in patients with ischemic stroke. 32 patients with acute or subacute infarcts detected by DSC imaging were reviewed. Each patient’s brain was divided into 12 areas. ASL ranks were defined by the presence (+) or absence (-) of PPD/HVS as follows; I:–/–, II:–/+, III: +/+, and IV: +/–. DSC imaging findings were categorized based on cerebral blood flow (CBF) and time to peak (TTP) as normal (normal CBF/TTP), mismatched (normal CBF/delayed TTP), and matched (decreased CBF/delayed TTP). Two reviewers rated perfusion abnormalities in the total of 384 areas. The four ASL ranks correlated well with the DSC subtypes (Spearman’s r = 0.82). The performance of ASL ranking system was excellent as indicated by the area under the curve value of 0.94 using either matched or mismatched DSC subtype as the gold standard and 0.97 using only the matched DSC subtype as the gold standard. The two methods were in good-to-excellent agreement (maximum κ-values, 0.86). Inter-observer agreement was excellent (κ-value, 0.98). Although the number of patients was small and the number of dropouts was high, our proposed, ASL-based visual ranking system represented by PPD and HVS provides good, graded estimates of perfusion disturbance that agree well with those obtained by DSC perfusion imaging.

## Introduction

Perfusion-weighted imaging (PWI) is a useful diagnostic tool in the clinical evaluation of ischemic stroke. Dynamic susceptibility contrast imaging (DSC) is an MR perfusion imaging technique performed with intravenous injection of a contrast agent and is widely used for patients with ischemic stroke. DSC imaging enables measurement of four parameters: cerebral blood volume (CBV), cerebral blood flow (CBF), time to peak (TTP), and mean transit time (MTT) and also allows the severity of the infarcts to be estimated [1]. The technique does have limitations, however, including adverse effects of the contrast agent, and the additional cost.

Arterial spin labeling imaging (ASL), another MR perfusion imaging technique, does not require an exogenous contrast agent. Because the technique is based on the arterial spin labeling of a freely diffusible tracer, ASL allows a quantitative CBF assessment [2]. Previous studies revealed that values calculated from ASL images in patients with acute ischemic stroke corresponded well with those calculated from DSC perfusion images, and that ASL images can also provide estimates of PWI-diffusion weighted imaging (DWI) mismatches [3–5]. Also, the parenchymal CBF values obtained by ASL imaging have been used to identify impaired cerebrovascular reactivity in patients with Moyamoya disease [6].

Some studies have reported that ASL imaging reveals vessel structures with high signal intensity if the post-labeling delay is less than the arterial arrival time due to a slow arterial flow rate. These high signal intensities observed using ASL imaging have been interpreted as indicating delayed arterial transit or stagnant flow proximal to the occlusion sites [4,7,8].

With the above background in mind, we hypothesized that hyperintense vessel signals (HVS) could serve as a potential indicator of perfusion delay and have developed a novel visual ranking system indicating the severity of perfusion abnormalities by combining two ASL imaging parameters, including the parenchymal signal as the indicator of blood flow and HVS. The aim of this study was to correlate this ASL-based ranking system with DSC-based subtypes which also indicate the severity ranks of the perfusion disturbance in patients with ischemic stroke.

## Materials and methods

### Patient cohort

Institutional Review Board on Human Subjects Research and Ethics Committees Hanyang University Guri Hospital approved this retrospective study, and informed consent was waived.

**Fig 1:**
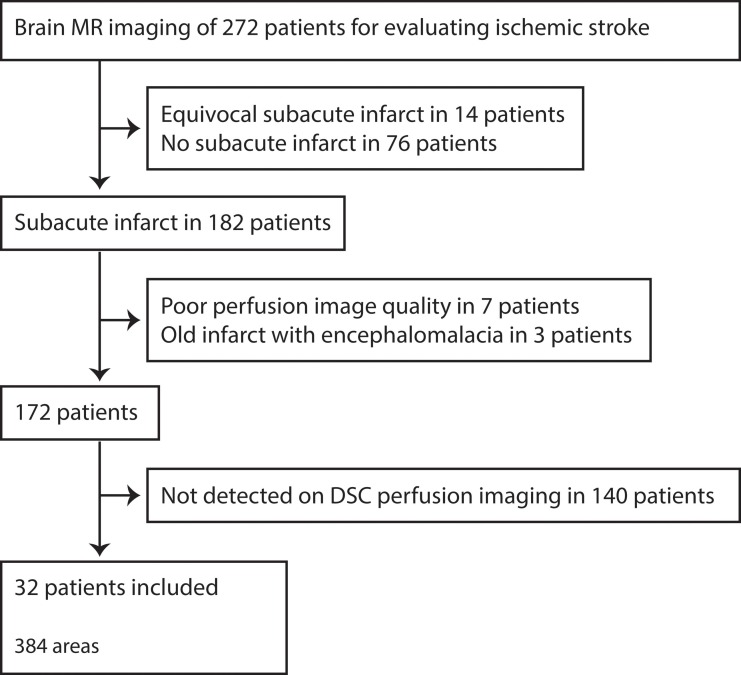
Flow chart of patients included in the study.

The hospital database was searched for patients who were admitted over a 6-month period with a presumptive clinical diagnosis of ischemic stroke and subsequently underwent brain MR imaging, including ASL imaging and DSC perfusion imaging. The search yielded 272 such patients. A board-certified neuroradiologist reviewed MR imaging. Of the 272 patients, 182 showed definite diffusion restriction, 14 had equivocal diffusivity restrictive lesions requiring clinical confirmation, and 76 had no evidence of infarct (Fig 1). Of the 182 patients with definite diffusion restriction, 10 patients were excluded; seven of the 10 patients were excluded due to the poor quality of the perfusion images (ASL images in three, DSC images in two, and both ASL and DSC images in two) resulting from patient motion, and three patients due to the presence of old infarcts and associated cortical encephalomalacia that can be confused with new infarcts during the perfusion image analysis. Of the remaining 172 patients, 140 had infarcts that were not seen on DSC perfusion imaging, and 32 had infarcts that were described as matched perfusion defects affecting both CBF and TTP, according to DSC perfusion imaging.

Of the 32 patients included in this study; 20 were men, and 12 were women. The median age was 70 years (range, 48–87 years). In eight of the 32 patients, the time of symptom onset was known (either witnessed or noticed by the patient); the median interval between the time of symptom onset and MR imaging was 31 hours (range, 14–75 hours). In 18 of the 32 patients, although the exact moment of symptom onset was not witnessed or noticed by the patient, the first abnormal time (the time when the patient was first found to have symptoms, e.g., ‘wake-up’ strokes) was known; the median interval between the first abnormal time and MR imaging was 31 hours (range, 11–197 hours). In the remaining 6 patients, none of these times were available in the database.

### Imaging modality and protocol

MR imaging was performed using a 3T MR scanner (Philips Healthcare, Best, the Netherlands) and a 32-channel head coil.

A pseudo-continuous ASL technique was used, during which the repetition of very short duration radiofrequency pulses produces effective arterial proton labeling [9]. The labeling plane was positioned 90 mm proximal to the imaging plane, labeling duration was 1,800 msec, and the post-label delay was 1,600 msec. Images were obtained using the following parameters: repetition time/echo time 5,000/18 msec; flip angle, 90°; field of view, 200 x 200 mm; acquisition matrix, 100 x 98 (m x p); voxel resolution, 2 x 2 x 6 mm; background suppression pulses, two inversion pulse for nulling static tissue signal intensity; and total scan time, 6 min 50 sec.

DSC perfusion images were obtained using T2*-weighted fast gradient-echo sequences (T2 FFE; Philips) after the intravenous administration of gadoterate meglumine (0.1 mmol/kg, standard dose; 3.5ml/sec, flow rate). Images were obtained using the following parameters: repetition time/echo time 2,950/35 msec; flip angle, 40°; field of view, 200 x 200 mm; acquisition matrix, 124 x 121; voxel resolution, 1.5 x 1.5 x 4.5 mm; dynamic, 50; and total scan time, 2 min 35 sec. Measurements of arterial input function were performed semi-automatically with commercial software (nordicICE; NordicNeuroLab, Bergen, Norway). For quality check, we confirmed that the steady states of signals were reached near the baseline.

There were 13 min 5 sec intervals between the ASL and DSC scans. The images were obtained parallel to the intercommissural line in both sequences. In addition, the following sequences were performed; DWI (B = 0, B = 1,000), apparent diffusion coefficient map, contrast-enhanced angiography of the extra- and intracranial vessels, time of flight angiography of the intracranial vessels, black blood turbo spin echo T1-weighted imaging before and after contrast enhancement, and fluid-attenuated inversion recovery. All the serial sequences were made in one sitting.

### Image analysis

ASL and DSC perfusion images were analyzed and compared, focusing on the presence and severity of the perfusion defects. Two board-certified neuroradiologists performed a randomized, independent, and blinded review of the images; they were not informed of the study details. Neither of the two neuroradiologists was the one who initially screened the 272 patients’ MRI. The DSC images were evaluated 3 months after the ASL images were evaluated, and the images were evaluated in random order.

**Fig 2:**
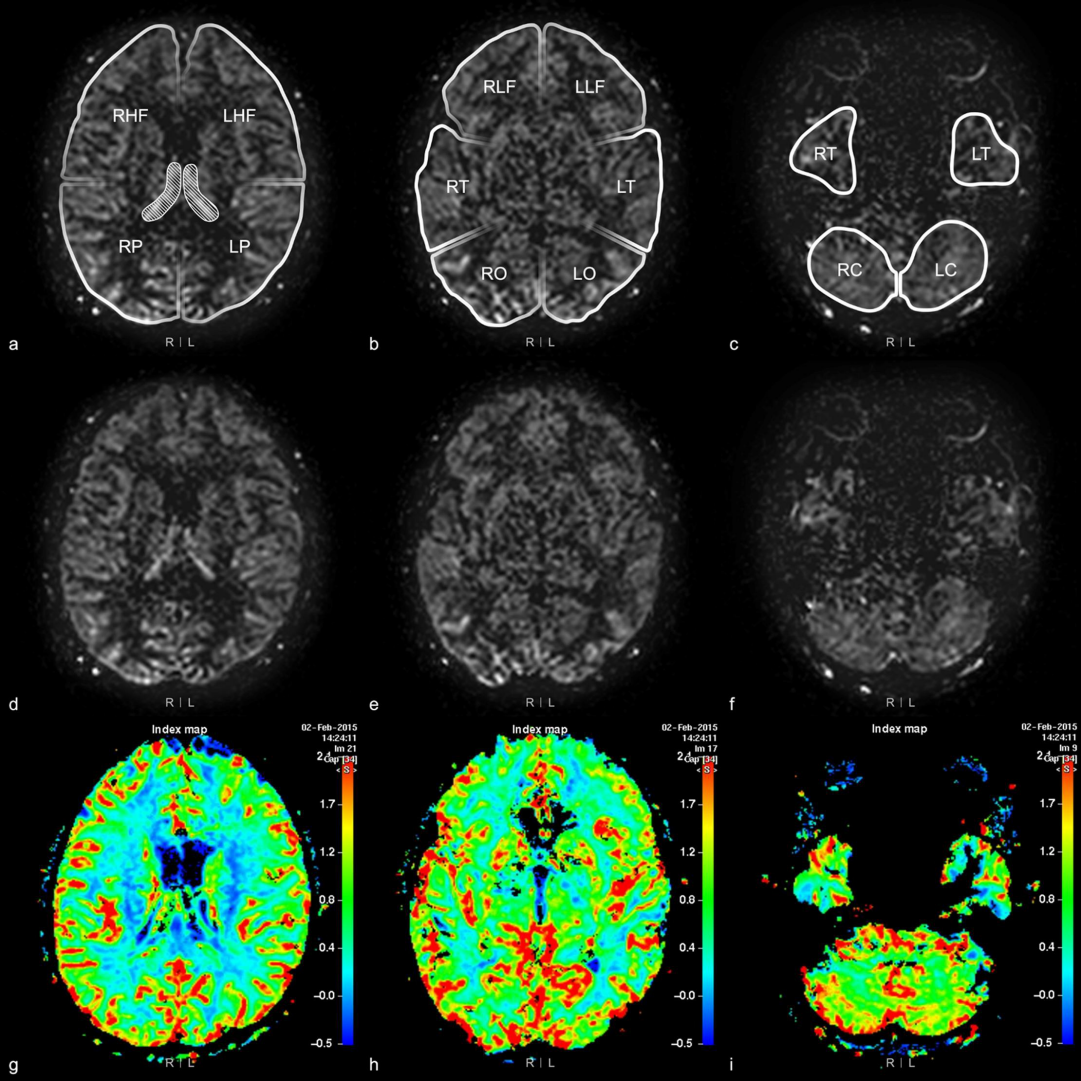
12 areas of bilateral cerebral and cerebellar hemispheres. Brain MRI of a 76-year-old woman with dizziness. 12 areas on arterial spin labeling images are shown in figures (a), (b), and (c). Figures (d), (e), and (f) are ASL images and figures (g), (h), and (i) are cerebral blood flow map. Figures (a), (d), and (g) show the most superior portion of the bilateral choroid plexus (regions marked by oblique lines in figure (a)). The image slices above the landmark, the choroid plexus in the trigone, are divided into right and left high frontal (RHF, LHF) and parietal (RP, LP) areas, and the lower image slices are divided into the other eight areas; the low frontal (RLF, LLF), temporal (RT, LT), occipital (RO, LO), and cerebellar (RC, LC) areas on both sides.

Bilateral cerebral and cerebellar hemispheres were divided into 12 areas: high frontal, low frontal, parietal, temporal, occipital, and cerebellar areas in each hemisphere. The uppermost image slice showing the choroid plexus in the trigone of the lateral ventricle was chosen as the landmark. The landmark slice and the image slices above this landmark were divided into the high frontal and parietal areas. The image slices below this landmark were divided into the low frontal, temporal, occipital and cerebellar areas (Fig 2). If there were severe perfusion abnormality (e.g., difficult to recognize the landmark) in the ipsilateral hemisphere, the anatomical location was recorded as the contralateral hemisphere.

**Fig 3:**
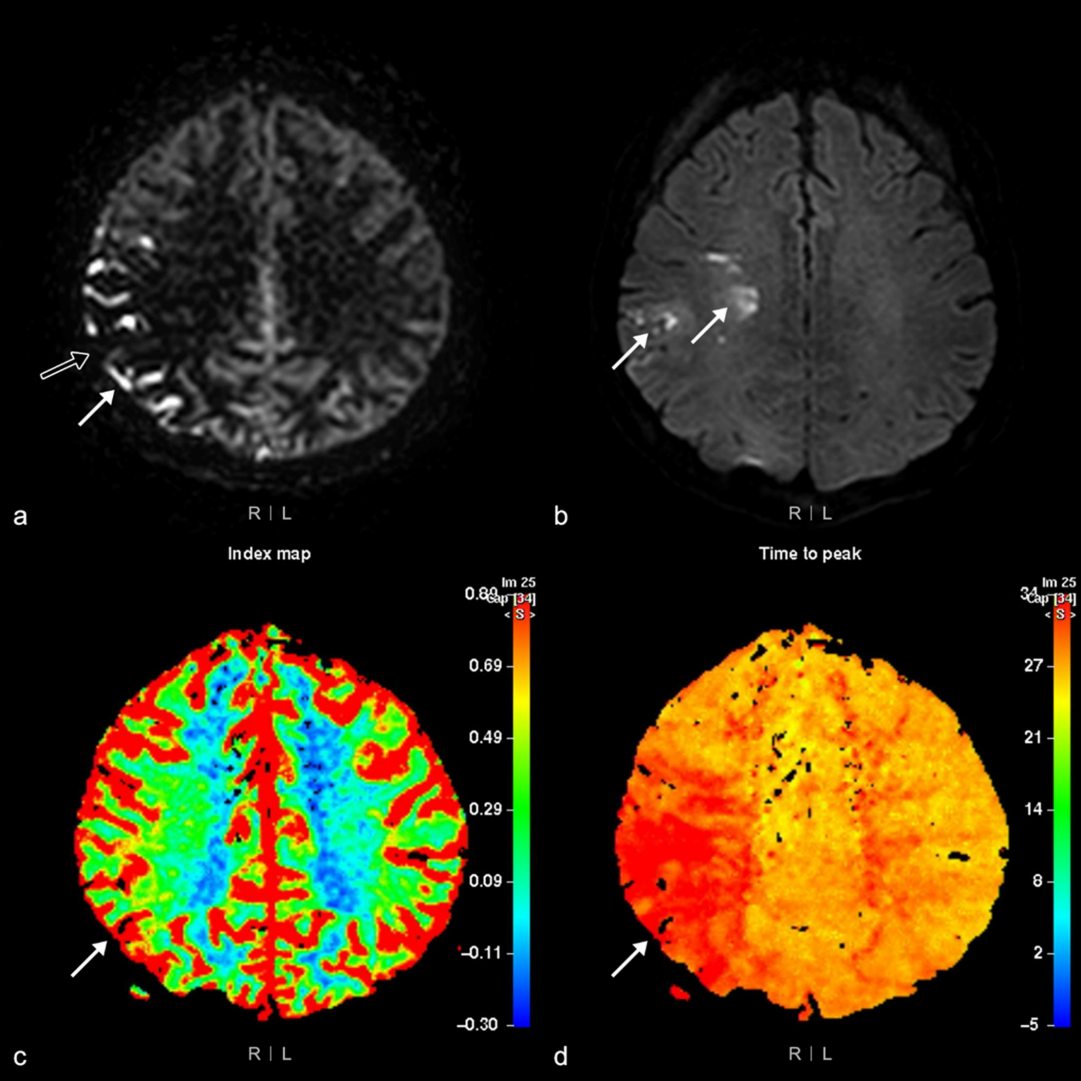
Arterial spin labeling (ASL) rank III perfusion abnormality. MRI of the brain of a 48-year-old woman with left-side motor weakness. The ASL image (a) shows a rank III perfusion abnormality, as hyperintense vessel signals (arrow) and parenchymal perfusion deficits (open arrow) are present in the right parietal area. However, high signal intensity (arrow) visualized by diffusion-weighted imaging (b = 1,000) (b) is detected only in the right frontal white matter and parietal area. Cerebral blood flow (c) and time to peak maps (d) show a mismatched perfusion defect (arrow) in the right parietal area.

**Fig 4:**
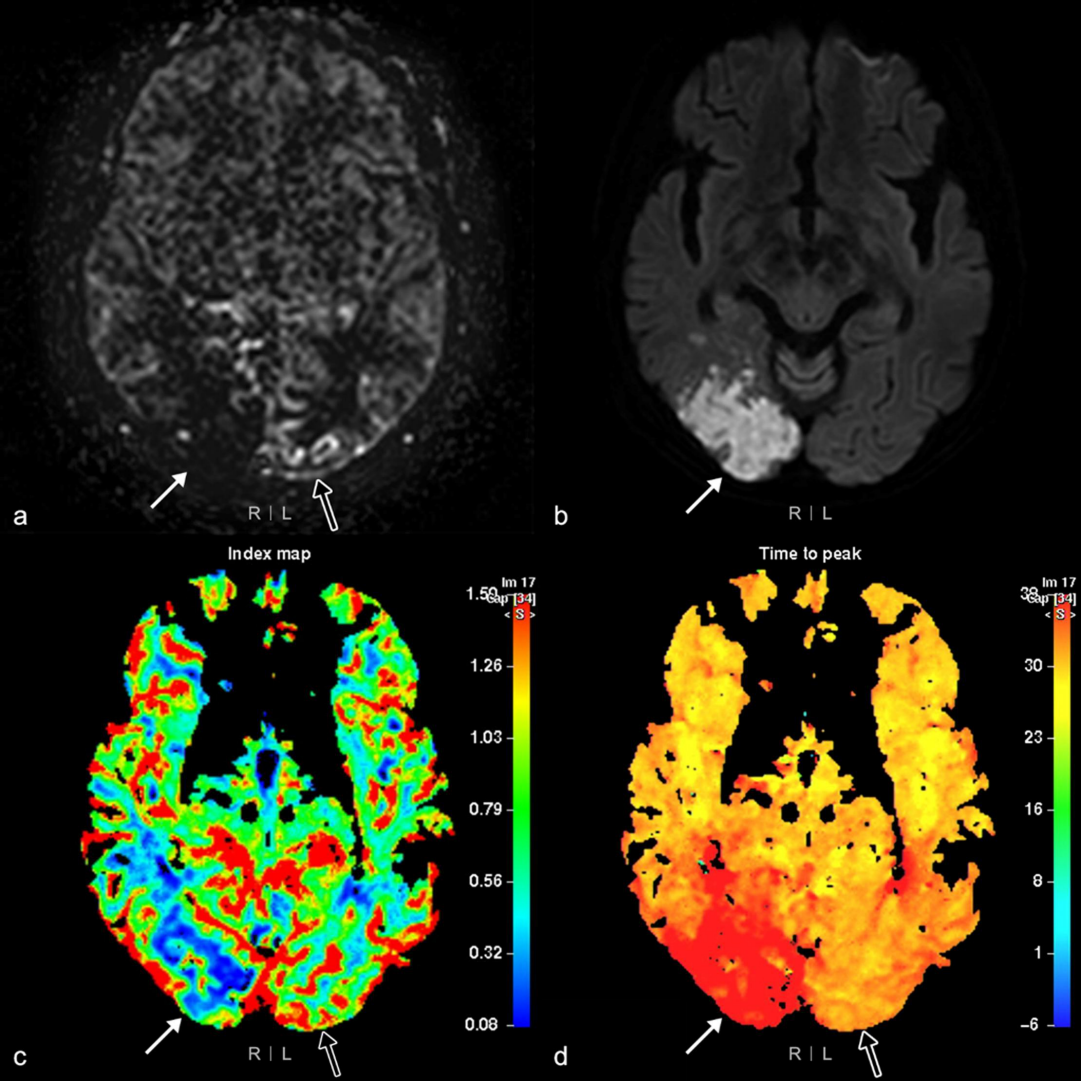
Arterial spin labeling (ASL) rank II and IV perfusion abnormalities. MRI of the brain of a 78-year-old woman with a left visual field defect. (a) ASL imaging shows a rank IV perfusion abnormality; positive parenchymal perfusion deficits (PPD) in the right occipital area (arrow); (b) Diffusion weighted imaging (b = 1,000) shows a high signal intensity (arrow); (c) cerebral blood flow (CBF) and (d) time to peak (TTP) maps show matched perfusion defects (arrow) in the same area. (a) A rank II perfusion abnormality is visualized as positive hyperintense vessel signals without PPD (open arrow) using ASL imaging, and a normal (open arrow) is seen in the left occipital area on (c) CBF and (d) TTP maps.

Perfusion abnormalities visualized by ASL imaging were categorized into four ranks by the two radiologists. The four ranks were assigned to each of the 12 areas, according to the presence or absence of parenchymal perfusion deficits (PPD+ or PPD-) and the presence or absence of hyperintense vessel signals (HVS+ or HVS-). As the white matter signal is inherently low on ASL imaging, PPD were graded as present or absent based on the cortical signal intensity. Each area was ranked as ASL-I if PPD-/HVS-, ASL-II if PPD-/HVS+, ASL-III if PPD+/HVS+, or ASL-IV if PPD+/HVS- (Figs 3 and 4). If two different ranks were identified within the same area simultaneously, the higher rank was chosen for data analysis.

To evaluate DSC perfusion images, we chose the CBF and TTP maps as perfusion parameters among four DSC perfusion maps. CBF was chosen because ASL is a noninvasive tool for measuring blood flow. Between TTP and MTT maps, TTP was chosen because it reflects the extent and location of the ischemic lesion relatively more accurately than the latter [2,10–12]. Findings in each of the 12 areas on the DSC images were categorized into one of three subtypes; normal, mismatched, and matched. Matched perfusion defects were defined as the presence of abnormal area on both the CBF and TTP maps. Mismatched perfusion defects denote the presence of abnormalities on the TTP map only (Figs 3 and 4). If there were two different subtypes identified in one area, the worse subtype was assigned to that area. For example, if both matched and mismatched perfusion defects were present in the same area, the area was recorded as having a matched perfusion defect; if both normal tissue and mismatched or matched perfusion defects were identified in the same area, the area was recorded as having a mismatched or matched perfusion defect. When there was discordance between the two radiologists, they reviewed the patient images together and reached a consensus.

After analyzing the ASL and DSC perfusion images, we reviewed other images mentioned in the imaging modality and protocol section to characterize the patient cohort.

### Statistical analyses

The statistical analyses were performed based on the area level. The correlation between ASL ranks and DSC subtypes was analyzed using Spearman’s rank correlation coefficient. To analyze the relationship between ASL ranks and DSC subtypes, receiver operating characteristic (ROC) curves, sensitivity, specificity, and positive and negative predictive values (PPV and NPV) were measured for the ASL images, using DSC perfusion imaging as the gold standard. Weighted Kappa values for the degree of agreement between ASL ranks and DSC subtypes were calculated. For the Kappa statistic, ASL ranks and DSC subtypes were re-classified as trichotomous or dichotomous groups. Additionally, subgroup analysis was performed using Fisher exact test for the patients with known time of symptom onset or first abnormal time.

A weighted Kappa value was also calculated to assess inter-observer agreement before coming to a consensus. Kappa values of<0.20, 0.21–0.40, 0.41–0.60, 0.61–0.80, and 0.81–1.00 were considered to represent a poor, fair, moderate, good, and excellent agreement, respectively. P values < 0.05 were considered statistically significant. Spearman’s rank correlation coefficient and Fisher exact test were calculated using SPSS, and ROC curves, diagnostic values, and weighted Kappa values were obtained using MedCalc.

## Results

### Infarct territories and vascular abnormalities

Of the 32 patients included in this study, the left middle cerebral artery (MCA) territory was the most frequent sites of infarcts, which was present in 14 patients. The right MCA territory was the second most frequent site of infarct found in 5 patients. Frequencies of infarcts in other vascular territories and those of more detailed vascular abnormalities within each territory are summarized in Table 1.

**Table 1:** Radiologic evaluation of infarcts in 32 patients.

Infarct territory (number of patients)	Vascular abnormalities on MRI* (number of patients)
Left MCA (14)	Left MCA (6) Left ICA (8)
Right MCA (5)	Right MCA (4) Right MCA(1) ^†^
Left PICA (3)	Left PICA (2) Left VA to BA (1)^†^
Watershed zone (3)	Left ICA (1) Left ICA, right MCA(1) Right ICA, both PCA (1)
Right PCA (2)	Right PCA (2)
Left PCA (1)	Left PCA (1)
Left ACA, MCA (1)	Left ACA, MCA (1)
Right MCA, PCA (1)	Right ICA^‡^ (1)
Bilateral PICA, right PCA (1)	Bilateral VA to BA^†^, right PCA(1)
Right PICA (1)	Right VA (1)^†^

ACA, anterior cerebral artery; BA, basilic artery; ICA, internal carotid artery; MCA, middle cerebral artery; PCA, posterior cerebral artery; PICA, posterior inferior cerebellar artery; VA, vertebral artery

* Vascular abnormalities on MR are occlusion or severe stenosis on MR angiography

^†^ Unstable plaque on black blood turbo spin echo T1-weighted imaging

^‡^ Right fetal type PCA on MR angiography

Twenty-eight patients had significant vascular narrowing, described as severe stenosis or occlusion on MR angiography, which correlated with the infarct area. Three of the remaining four patients had vascular wall enhancement, suggesting the presence of unstable plaque related to the infarct area, and one had vascular occlusion and unstable plaque (Table 1).

### ASL ranks and DSC subtypes

**Fig 5:**
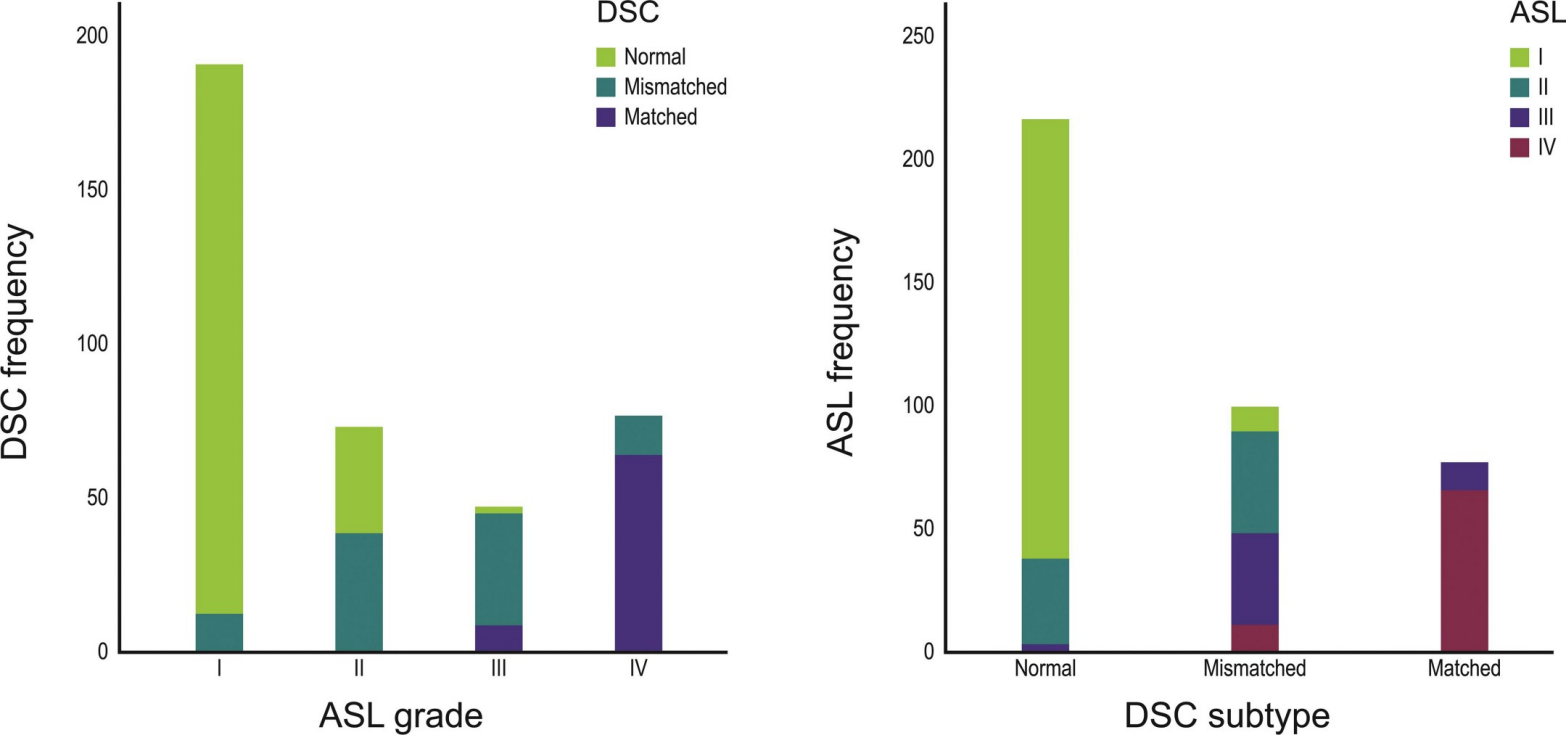
Distribution of the 384 areas showing perfusion abnormalities, visualized by arterial spin labeling (ASL) imaging and dynamic susceptibility (DSC) perfusion imaging, in the 32 patients with ischemic stroke (r = 0.82, p- = 0.01 by Spearman’s rank test). ASL-I refers to no PPD or HVS, II refers to no PPD with the presence of HVS, III refers to the presence of both HVS and PPD, and IV refers to the presence of PPD without HVS.

Of the 384 areas in the 32 patients, 190 were ranked as I by ASL imaging. The vast majority (n = 178) of these 190 areas were normal on DSC imaging (93.7%), 12 had mismatched perfusion abnormalities (6.3%), and none of the areas had a matched perfusion abnormality. Seventy-two areas ranked as II on ASL imaging were either normal or ‘mismatched’ on DSC imaging; none were matched. The majority of the areas ranked as ASL-III were ‘mismatched’ on DSC imaging, and the majority of ASL-IV areas were ‘matched’ on DSC imaging. More detailed data are presented in Table 2 and Fig 5. Spearman’s rank correlation coefficient of the ranks determined by ASL imaging and subtypes determined by DSC perfusion imaging was 0.82 (p-value = 0.01).

**Table 2:** Numbers of areas showing perfusion abnormalities in 32 patients with ischemic stroke (with percentages of the proportions of each ASL rank and each DSC subtype within parentheses), as determined by ASL imaging and DSC perfusion imaging. ASL rank I refers to no HVS or PPD, II refers to the presence of HVS without PPD, III refers to the presence of HVS and PPD, and IV refers to the presence of PPD without HVS (r = 0.82, p = 0.01 by Spearman’s rank test).

	Rank	ASL				Total number
	Subtype	I	II	III	IV	
DSC	Normal	178 (93.7, 83.6)	33 (45.8, 15.5)	2 (4.3, 0.9)	0 (0.0, 0.0)	213 (100)
	Mismatched	12 (6.3, 12.2)	39 (54.2, 39.8)	36 (76.6, 36.7)	11 (14.7, 11.2)	98 (100)
	Matched	0 (0.0, 0.0)	0 (0.0, 0.0)	9 (19.1, 12.3)	64 (85.3, 87.7)	73 (100)
Total number		190 (100)	72 (100)	47 (100)	75 (100)	384

ASL, arterial spin labeling; DSC, dynamic susceptibility contrast; HVS, hyperintense vessel signals; PPD, parenchymal perfusion deficits

**Fig 6:**
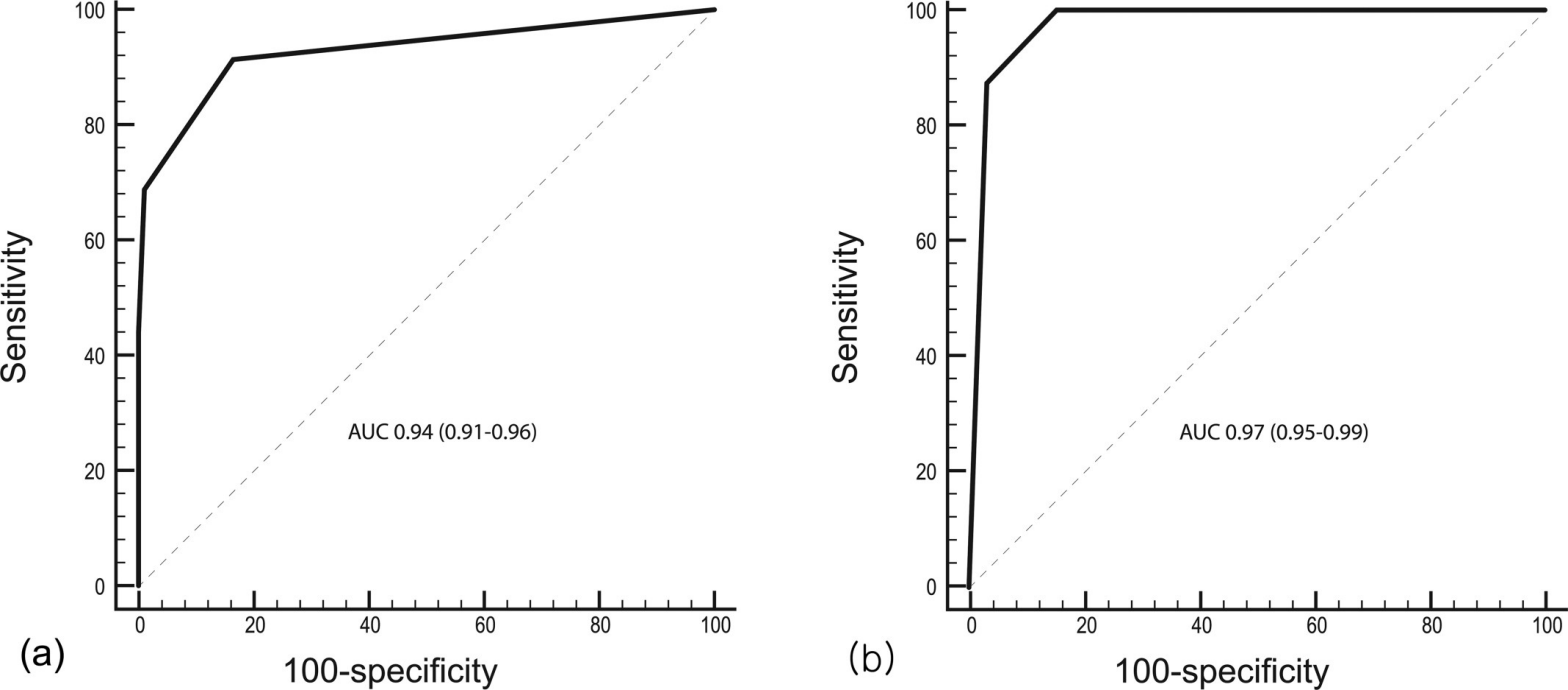
Receiver operating characteristic curves of the ranking system for arterial spin labeling (ASL) images, using either matched or mismatched perfusion defects (a) on dynamic susceptibility contrast (DSC) perfusion imaging or matched perfusion defects only (b) as the gold standard. The area under the curve (AUC) value was 0.94, and 0.97, respectively. Numbers within parentheses are 95% confidence interval.

When a positive diagnosis was defined as either matched or mismatched DSC perfusion imaging subtypes, the area under the curve (AUC) was 0.94 (95% confidence interval, 0.91–0.96). When a positive diagnosis was defined as matched perfusion defects alone, the AUC was 0.97 (95% confidence interval, 0.95–0.99). Additional data on the diagnostic sensitivity, specificity, PPV, and NPV are given in Fig 6 and Table 3. The weighted Kappa values between the ASL ranks and DSC subtypes were good-to-excellent in both trichotomous and dichotomous group comparisons (Table 4).

**Table 3:** Diagnostic performance of the ASL ranking system, using DSC perfusion imaging as the gold standard.

Criteria of ASL ranks	Sensitivity	Specificity	PPV	NPV
	a. Either matched or mismatched perfusion defect on DSC perfusion imaging as the gold standard			
≥I	100.0	0.0	44.5	-
≥II*	93.0	83.6	82.0	93.7
≥III	70.2	99.1	98.4	80.5
≥IV	43.9	100.0	100.0	68.9
>IV	0.0	100.0	-	55.5
	b. Matched perfusion defect on DSC perfusion imaging as the gold standard			
≥I	100.0	0.0	19.0	-
≥II	100.0	61.1	37.6	100.0
≥III*	100.0	84.2	59.8	100.0
≥IV	87.7	96.5	85.3	97.1
>IV	0.0	100.0	-	81.0

ASL, arterial spin labeling; DSC, dynamic susceptibility contrast; NPV, negative predictive value; PPV, positive predictive value

* maximum of the Youden index

**Table 4:** Kappa values of the trichotomous and dichotomous groups described by ASL ranks and DSC subtypes.

ASL	DSC	Kappa value
Rank I	Normal	0.82(0.78–0.86)*
Rank II	Mismatched	
Rank III, IV	Matched	
Rank I	Normal	0.86(0.82–0.89) *
Rank II, III	Mismatched	
Rank IV	Matched	
Rank I	Normal	0.76(0.69–0.82) *
Rank II, III, IV	Mismatched, matched	
Rank I, II, III	Normal, mismatched	0.83(0.76–0.90) *
Rank IV	Matched	

ASL, arterial spin labeling; DSC, dynamic susceptibility contrast

*Numbers within brackets are 95% confidence interval of the Kappa value

We further analyzed the ASL-II areas in 26 patients with the known first abnormal time (n = 18) or the time of symptom onset (n = 8). These patients were divided into two subgroups according to the interval between the first abnormal time or the symptom onset time and MR imaging (collectively abbreviated as the ‘Stroke-MR interval’ hereafter). The Stroke-MR interval was equal to or less than 24 hours in 11 patients and more than 24 hours in 15 patients. In the first subgroup of 11 patients (≤24 hours), there were 15 ASL-II areas of which, one was normal, and 14 were ‘mismatched’ on DSC perfusion imaging. Of the 48 ASL-II areas in the latter subgroup (> 24 hours), 28 were normal, and 20 were mismatched perfusion defect. The difference between the two groups was highly significant (2-tailed P value = 0.001).

Discrepancies in the interpretation of ASL imaging between the two observers were found only in 18 of the 384 areas and in 14 of the 384 areas in the interpretation of DSC imaging. Weighted Kappa values were 0.98 (95% confidence interval, 0.97–0.99) for ASL imaging and 0.97 (95% confidence interval, 0.95–0.99) for DSC perfusion imaging, indicating excellent inter-observer agreement for both ASL imaging and DSC perfusion imaging.

## Discussion

HVS on ASL imaging is reported to reflect delayed arterial transit [4] or stagnant flow proximal to the occlusion sites [7,8]. Based on the assumption that information provided by HVS would be related to that from TTP on DSC imaging, we developed an ASL-based ranking system by combining PPD with HVS to correlate with a DSC-based ranking system which is also based on a combination of two parameters indicating similar perfusion properties, i.e., CBF and TTP. Many studies have previously compared ASL imaging with DSC imaging qualitatively and/or quantitatively [3,5,13,14]. There were also more modern ASL sequence to be acquired several delay times that could give more objective perfusion parameters correlated with DSC or CT perfusion imaging, but it requires longer scan time [15, 16]. We attempted to develop a simple and reproducible, qualitative ranking system that can be more easily used in daily practice as quantitative analysis is generally labor-intensive and time-consuming, thus impractical. Also, while some of the above-mentioned authors focused only on infarcted regions in their studies [13,14], we assessed hemodynamic changes in the entire brain rather than only infarcted areas. Our results may be useful for initial evaluation of ischemic stroke patients because of the short acquisition time compared to ASL imaging to be acquired several delay times, no need for post-processing for quantitative analysis, and simple visual assessment algorithm (S1 Algorithm).

Our results showed that the four ASL-derived ranks of the perfusion status agreed quite well with the three subtypes (i.e., ranks) determined by DSC perfusion imaging with excellent kappa values. Most normal areas on DSC imaging corresponded to ASL-I, while most mismatched perfusion defects on DSC imaging were either ASL-II or ASL-III, and the vast majority of the matched defects on DSC imaging were ASL-IV.

Of the four ASL ranks, ASL-II showed the highest degree of disagreement with DSC imaging findings; only 54% (39/72) of ASL-II areas correlated with mismatched perfusion abnormalities on DSC imaging, and the remaining 46% (33/72) of ASL-II areas were normal on DSC imaging. These results suggest that ASL-II may overrate the degree of perfusion abnormalities, as shown in Table 2 and Fig 5. Possible explanations include only HVS without PPD being defined as perfusion abnormalities on ASL imaging and the post-label delay time of 1600 msec that might have been short in individual patients. At the same time, it is also possible that DSC imaging underestimates the severity of infarct in some of the areas showing ASL-II and normal DSC. Interestingly, our subgroup analysis showed that only one of 15 ASL-II areas in patients with a Stroke-MR interval of ≤ 24 hours had normal DSC, whereas 28 of 48 ASL-II areas in patients with a Stroke-MR interval of > 24 hours had normal DSC. In other words, all but one (28/29) areas with ASL-II and normal DSC were observed in patients with the Stroke-MR interval of > 24 hours. Given these results, it seems quite reasonable to presume that luxury perfusion could be one of the explanations of ASL-II with normal DSC. While further studies will be needed to clarify which one of the above listed possible explanations are true and/or more important, the Stroke-MR interval appears to be an important factor to be considered in the interpretation of ASL-II finding.

There were some limitations in the current study. Given its retrospective nature, the presence of sampling bias cannot be ruled out. Our findings were based on small number of patients only and that there was a high number of dropout patients due to missing DSC perfusion which raises a high suspicion of selection bias. Missing CBF quantification is a major limitation as no precise comparison of CBF between ASL and DSC was possible in our study. In addition, our departmental protocol was to use a post-labeling delay of 1,600 msec for ASL imaging, which could have resulted in ASL-II in some brain areas as discussed above. The author of the previous paper [17] described 2000 msec post-labeling delay as the most appropriate for CBF quantification for the clinical adult population, but before proceeding with this study, we performed a visual assessment by applying various post-labeling delays in our center and then selected 1600 msec as the most appropriate for visual assessment. Although a 1600 msec delay did not prevent the hyperintense spot, we could split the grade II and III by analyzing this spot and parenchymal signal together, and it is clear that at least in our proposed algorithm, grade II is better perfusion status than grade III. If we develop the research using CBF quantification in the future, it seems necessary to adjust the post-labeling delay and to analyze the ASL image base on the calculated CBF value. Despite these limitations, the statistical results were strong. Further, an inter-observer agreement for interpretation of ASL imaging was very high with a weighted kappa value of 0.98. These results indicate that the new visual ranking system is objective and reproducible. For these reasons, we believe that our new visual ranking system can be potentially valuable and practical and that future researchesare warranted in a larger cohort with ischemic stroke.

## Conclusions

This preliminary study suggests that our proposed, ASL-based visual ranking system represented by parenchymal perfusion deficits and hyperintense vessel signals provides good, graded estimates of perfusion disturbance that correspond to those assessed by DSC perfusion imaging with a high degree of agreement. Further studies are warranted to validate our results in a larger patient cohort and to assess the precise clinical impact of this ranking.

## Supporting information

S1 Algorithm (PDF)

## References

[1] Baird AE, Warach S. Magnetic resonance imaging of acute stroke. J Cereb Blood Flow Metab. 1998;18:583–609. 10.1097/00004647-199806000-00001 9626183

[2] Alsop DC, Detre JA. Multisection cerebral blood flow MR imaging with continuous arterial spin labeling. Radiology. 1998;208:410–416. 10.1148/radiology.208.2.9680569 9680569

[3] Niibo T, Ohta H, Yonenaga K, Ikushima I, Miyata S, Takeshima H. Arterial spin-labeled perfusion imaging to predict mismatch in acute ischemic stroke. Stroke. 2013;44:2601–2603. 10.1161/STROKEAHA.113.002097 23868269

[4] Chalela JA, Alsop DC, Gonzalez-Atavales JB, Maldjian JA, Kasner SE, Detre JA. Magnetic resonance perfusion imaging in acute ischemic stroke using continuous arterial spin labeling. Stroke. 2000;31:680–687. 10.1161/01.str.31.3.680 10700504

[5] Wang DJ, Alger JR, Qiao JX, Hao Q, Hou S, Fiaz R, et al The value of arterial spin-labeled perfusion imaging in acute ischemic stroke: comparison with dynamic susceptibility contrast-enhanced MRI. Stroke. 2012;43:1018–1024. 10.1161/STROKEAHA.111.631929 22328551 PMC3314714

[6] Yun TJ, Paeng JC, Sohn CH, Kim JE, Kang HS, Yoon BW, et al Monitoring Cerebrovascular Reactivity through the Use of Arterial Spin Labeling in Patients with Moyamoya Disease. Radiology. 2016;278:205–213. 10.1148/radiol.2015141865 26197057

[7] Tada Y, Satomi J, Abe T, Kuwayama K, Sogabe S, Fujita K, et al Intra-arterial signal on arterial spin labeling perfusion MRI to identify the presence of acute middle cerebral artery occlusion. Cerebrovasc Dis. 2014;38:191–196. 10.1159/000365653 25300901

[8] Yoo RE, Yun TJ, Rhim JH, Yoon BW, Kang KM, Choi SH, et al Bright vessel appearance on arterial spin labeling MRI for localizing arterial occlusion in acute ischemic stroke. Stroke. 2015;46:564–567. 10.1161/STROKEAHA.114.007797 25523057

[9] Zaharchuk G, Bammer R, Straka M, Shankaranarayan A, Alsop DC, Fischbein NJ, et al Arterial spin-label imaging in patients with normal bolus perfusion-weighted MR imaging findings: pilot identification of the borderzone sign. Radiology. 2009;252:797–807. 10.1148/radiol.2523082018 19703858 PMC6939961

[10] Detre JA, Alsop DC, Vives LR, Maccotta L, Teener JW, Raps EC. Noninvasive MRI evaluation of cerebral blood flow in cerebrovascular disease. Neurology. 1998;50:633–641. 10.1212/wnl.50.3.633 9521248

[11] Wittsack HJ, Ritzl A, Fink GR, Wenserski F, Siebler M, Seitz RJ, et al MR imaging in acute stroke: diffusion-weighted and perfusion imaging parameters for predicting infarct size. Radiology. 2002;222:397–403. 10.1148/radiol.2222001731 11818605

[12] Nael K, Meshksar A, Liebeskind DS, Coull BM, Krupinski EA, Villablanca JP. Quantitative analysis of hypoperfusion in acute stroke: arterial spin labeling versus dynamic susceptibility contrast. Stroke. 2013;44:3090–3096. 10.1161/STROKEAHA.113.002377 23988646 PMC4160882

[13] Huck S, Kerl HU, Al-Zghloul M, Groden C, Nolte I. Arterial spin labeling at 3.0 Tesla in subacute ischemia: comparison to dynamic susceptibility perfusion. Clin neuroradiol. 2012;22:29–37. 10.1007/s00062-011-0126-x 22270833

[14] Bokkers RP, Hernandez DA, Merino JG, Mirasol RV, van Osch MJ, Hendrikse J, et al Whole-brain arterial spin labeling perfusion MRI in patients with acute stroke. Stroke. 2012;43:1290–1294. 10.1161/STROKEAHA.110.589234 22426319 PMC3336029

[15] Wang DJ, Alger JR, Qiao JX, Gunther M, Pope WB, Saver JL, et al Multi-delay multi-parametric arterial spin-labeled perfusion MRI in acute ischemic stroke—Comparison with dynamic susceptibility contrast enhanced perfusion imaging. Neuroimage Clin. 2013;3:1–7. 10.1016/j.nicl.2013.06.017 24159561 PMC3791289

[16] Wang R, Yu S, Alger JR, Zuo Z, Chen J, Wang R, et al Multi-delay Arterial Spin Labeling Perfusion MRI in Moyamoya Disease—Comparison with CT Perfusion Imaging. Eur Radiol. 2014;24:1135–1144. 10.1007/s00330-014-3098-9 24557051 PMC4143230

[17] Alsop DC, Detre JA, Golay X, Gunther M, Hendrikse J, Hernandez-Garcia L, et al Recommended implementation of arterial spin-labeled perfusion MRI for clinical applications: A consensus of the ISMRM perfusion study group and the European consortium for ASL in dementia. Magn Reson Med. 2015;73:102–116. 10.1002/mrm.25197 24715426 PMC4190138

