# Supplementary material for: A novel visual ranking system based on arterial spin labeling perfusion imaging for evaluating perfusion disturbance in patients with ischemic stroke
Source: PLoS One. 2020 Jan 24;15(1):e0227747. doi: 10.1371/journal.pone.0227747 (PMC6980418; doi:10.1371/journal.pone.0227747)
Supplement: S1 Algorithm — (PDF) [file pone.0227747.s001.pdf]

Supporting information – algorithm of visual ranking system based on arterial spin labeling perfusion imaging for evaluating perfusion disturbance in patients with ischemic stroke (1600msec post-label delay time)

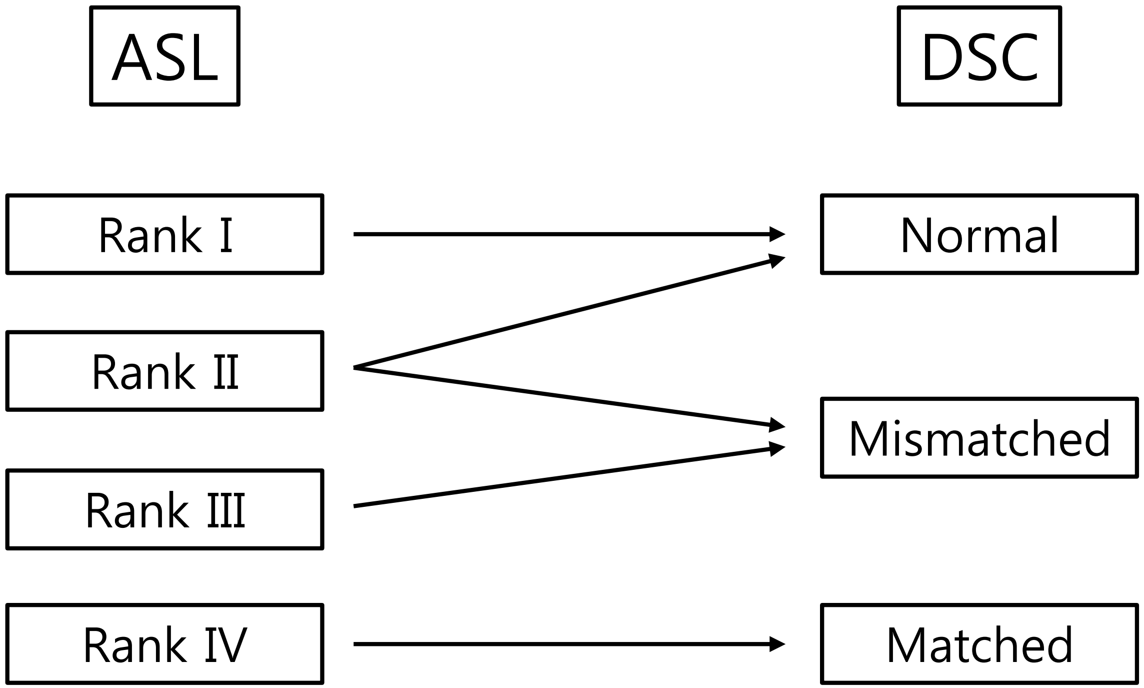

ASL imaging		
**Rank**	HVS	PPD
**I**	-	-
**II**	+	-
**III**	+	+
**IV**	-	+

DSC perfusion imaging		
**Subtype**	CBF	TTP
**Normal**	No decrease	No delay
**Mismatched**	No decrease	Delay
**Matched**	Decrease	Delay

ASL, arterial spin labeling; HVS, hyperintense vessel signals; PPD, parenchymal perfusion deficits;

DSC, dynamic susceptibility contrast; CBF, cerebral blood flow; TTP, time to peak
